# Assessing the Aesthetic Performance of CAD/CAM Provisional Restorative Materials: A Spectrophotometric Analysis

**DOI:** 10.3390/polym16182636

**Published:** 2024-09-18

**Authors:** Sónia Silvério, Catarina Gomes, Francisco Martins, José Alexandre Reis, Paulo Durão Maurício, José Eduardo Maté-Sánchez de Val

**Affiliations:** 1 Health Sciences PhD Program, Universidad Católica de Murcia UCAM, Campus de los Jerónimos n°135, Guadalupe, 30107 Murcia, Spain; ssilverio@egasmoniz.edu.pt (S.S.); jemate@ucam.edu (J.E.M.-S.d.V.); 2 Egas Moniz Center for Interdisciplinary Research (CiiEM), Egas Moniz School of Health & Science, Campus Universitário, Quinta da Granja, 2829-511 Almada, Portugalfmartins@egasmoniz.edu.pt (F.M.);; 3 Department of Materials Science and Engineering, International Research Institute for Biomaterials, Faculty of Health Sciences, Universidad Católica de Murcia UCAM, 30107 Murcia, Spain

**Keywords:** provisionals, dental veneers, spectrophotometry, dental materials

## Abstract

Achieving color match between natural teeth and restorative materials is crucial in dentistry. Factors such as the light source, brightness, and opacity influence tooth color, determined by light absorption and scattering within the material. Advances in CAD/CAM systems have enhanced prosthodontic treatments, particularly with new temporary materials, but data on their color stability and masking ability remains limited. However, data on the color stability and masking ability of these CAD/CAM materials is limited. Telio^®^ CAD-Temp and VITA CAD-Temp^®^ blocks were cut into slices and polished. Composite resin specimens were prepared using a custom-designed metal resin former and light-cured. Samples were paired randomly and assigned to experimental groups based on base type and thickness (*n* = 30). Samples were stored in a controlled environment for 24 h before color evaluation using an EasyShade^®^ V spectrophotometer. Color difference (Δ*E*) was calculated using L*, a*, and b* values. Statistical analyses included the Shapiro–Wilk test, Levene’s test, and three-way ANOVA, with post-hoc comparisons using the Bonferroni method (α = 0.05). Δ*E* was classified according to perceptibility (PT = 1.2) and acceptability (AT = 2.7) values.

## 1. Introduction

Teeth colour is a phenomenon influenced by primary and secondary optical properties, and it is determined by the interaction of various components such as brightness, light source, opacity, and the observer’s visual perception [1]. The color match between natural teeth and restorative materials is one of the most important and most difficult tasks in dentistry from the point of view of aestheticism [2].

Tooth color is a visual perception that is affected by several optical characteristics such as light diffraction, lighting environment/light source, transparency, opacity, and gloss, as well as visual information processing and brain control [3,4]. A person with naturally yellow teeth may perceive their teeth to be more yellow than a person with naturally white teeth, even if the actual color of their teeth is the same [5].

The peculiar color of dental structures is defined by the absorption and diffusion (scattering) of light within the tooth material [6]. While the clear enamel may not totally conceal the underlying dentin color and contributes to the absorbance of blue wavelengths itself, it is the dentin color that determines the shade of the tooth in the end [1,7,8].

The shift towards CAD/CAM systems in fixed prosthodontics has led to significant advancements in treatment approaches in glass-ceramic restorations, demonstrating long-term clinical success [9]. Nonetheless, the introduction of new CAD/CAM temporary materials in prosthodontics in the past few years has opened the door to new solutions that assure clinician success.

Adding to these properties, we also have the thickness, material type, and layering combination of ceramics contribute to the final restoration’s color. The selection of appropriate thickness plays a crucial role in achieving optimal aesthetic outcomes. A thin layer has limited masking ability, allowing the color of the underlying tooth structure and cement to show through [10]. Several studies have demonstrated the substantial impact of ceramic thickness on the final shade of restorations [11,12,13,14]. However, there is a lack of data regarding the use of temporary materials made by CAD/CAM.

Temporary fixed dental prostheses fabricated using indirect techniques can remain in service for up to two years [15,16]. This extended duration is particularly beneficial in clinical settings where alterations in the vertical or horizontal dimension of occlusion are necessary. In such cases, the use of long-term temporary restorations allows for a prolonged simulation period, which is essential for ensuring the stability and functionality of the occlusion adjustments [17,18]. This approach not only provides ample time to assess the patient’s adaptation to the changes but also ensures that the final restoration will meet the desired clinical and aesthetic outcomes [17]. Consequently, incorporating long-term temporary restorations into the treatment plan is mandatory for achieving optimal results in complex dental procedures.

By thoroughly understanding the importance of width and meticulously selecting the appropriate thickness for both provisional and definitive materials, clinicians can significantly enhance the overall aesthetic outcome of dental restorations. This careful consideration ensures that the final appearance is not only natural-looking but also harmonious with the patient’s existing dentition, leading to improved satisfaction and long-term success.

There is a lack of comprehensive data on the color stability and masking ability of these CAD/CAM provisional materials, especially when used with different substrate colors and thicknesses. Existing studies have primarily focused on permanent restorative materials, leaving a critical gap in understanding how temporary restorations perform in terms of aesthetics under varying clinical conditions [19,20,21,22]. This gap is particularly important given the increasing demand for aesthetic outcomes in dentistry, where even slight color mismatches can lead to patient dissatisfaction. This study aims to fill this gap by systematically evaluating the color stability and masking ability of two commonly used CAD/CAM provisional materials (Telio^®^ CAD-Temp and VITA CAD-Temp^®^) across different thicknesses (0.5 mm, 1.0 mm, and 2.0 mm) and substrate colors (A2 and A3). By employing a three-way mixed ANOVA to analyze the color differences (Δ*E_ab_*), this research seeks to determine how these variables interact and influence the aesthetic outcome of provisional restorations. The findings from this study will provide clinicians with evidence-based guidance on selecting appropriate materials and thicknesses to achieve optimal color matching, ultimately improving patient satisfaction and clinical success in dental practices.

The null hypothesis is that there is no significant difference in the color stability between different dental materials, regardless of the resin composite base, thickness, and type of material used.

## 2. Materials and Methods

Telio^®^ CAD-Temp (Ivoclar Vivadent; Schaan, Liechtenstein, Germany) and VITA CAD-Temp^®^ (VITA Zahnfabrik; Bad Säckingen, Germany) blocks were cut in slices with a Powermill Kondia (Clausing, MI, USA) Type FV-300 (serial number: S-913) with an IsoMetTM Diamond Wafering Brades diamond disc (Buehler^®^, Lake Bluff, IL, USA) 127 mm in diameter and 0.4 mm thick at 240 rpm, cooled with a mixture of 95% deionized water and 5% oil. One hundred and twenty samples were obtained in three different widths: 0.5, 1.0, and 2.0 mm. Each sample was 15.5 × 19 mm.

A LabolPol-4 (Stuers, Cleveland, OH, USA) grinding machine with sequential grinding papers (Carbimet 2; Buehler, Lake Bluff, IL, USA) of ISO/FEPA 500, 1000, and 1500 grit was used for polishing both sides of the samples at a constant speed of 100 rpm, for 15 s each.

To standardize the background, two shades, A2 and A3, of resin composite (Filtek^TM^ Supreme XTE Universal Restorative Body; 3M ESPE, St. Paul, MN, USA) (Table 1) were made. The sample thickness was 1 mm with a diameter of 14 mm. They were produced with a custom-designed metal resin former. And light-cured for 40 s at a high intensity (1000 mW/cm^2^), using an Elipar^TM^ (3M, St. Paul, MN, USA) LED light.

Polymers underwent a process of randomization and were assigned to the experimental groups according to the base, type, and thickness (*n* = 30).

The samples were stored in a dark, dry room temperature for 24 h.

**Figure 1:**
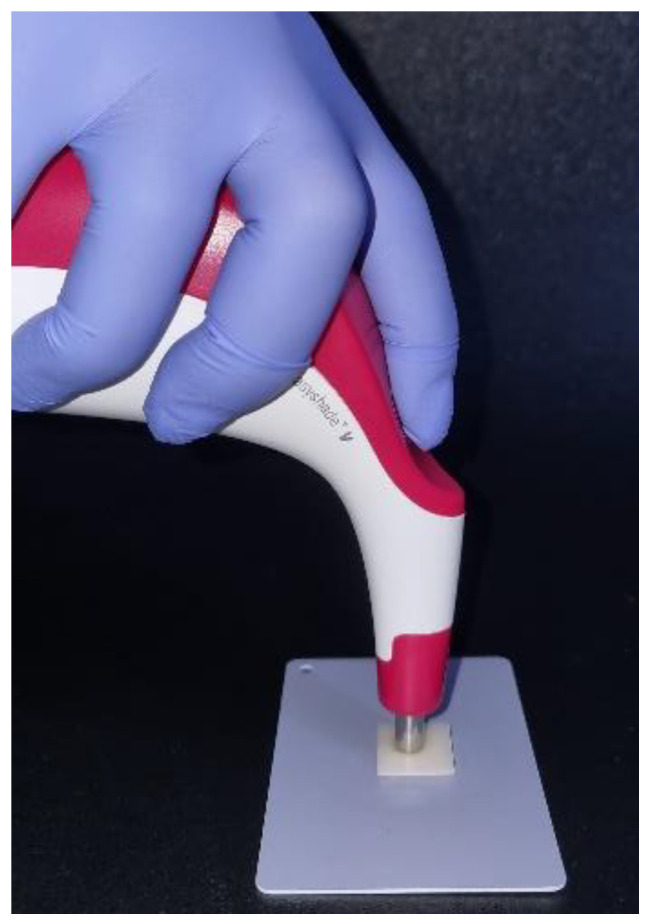
Colour measurement of a Telio sample on a grey background with the EasyShade V spectrophotometer.

The color was evaluated with an EasyShade V spectrophotometer (VITA Zahnfabrik, Bad Säckingen, Germany). The measurements were made in the L*, a*, and b* color space. The spectrophotometer was calibrated according to the manufacturer’s instructions, and color measurement was performed at the center of each sample on a gray background (Figure 1) in accordance with ISO/TR 28642:2016 [23,24]. Each material was measured two times, alone and then over the resin background.

Colour difference (Δ*E*) was calculated from the L*, a*, and b* values measured with the spectrophotometer. A grey background was used to standardize the measurements. According to the equation (below), the color difference (Δ*E*) was calculated [24]:

$$∆E=\sqrt{{\left(∆L^{*}\right)}^{2}+{\left(∆a^{*}\right)}^{2}+{\left(∆b^{*}\right)}^{2}}$$

The Shapiro–Wilk test showed the normal distribution, and the Levene test showed the homogeneity of variances. A three-way mixed ANOVA was performed to evaluate the interaction between the resin base, the thickness (0.5, 1.0, and 2.0 mm), and the type of provisional on the L*, a*, b*, and Δ*E_ab_* measurements. Post-hoc comparisons were performed using a Bonferroni method with a 95% confidence interval. All Statistical tests were performed with a statistical software program (IBM SPSS v28; IBM Corp., New York, NY, USA) (Δ = 0.05).

Color difference (Δ*E*) was also classified according to the most used perceptibility values, currently corresponding to a 50:50% perceptibility value (PT) of 1.2 and a 50:50% acceptability value (AT) of 2.7 [19,20,21,22].

## 3. Results

Presented in Table 2 are the Δ*E_ab_* mean values and standard deviations (SD) for shade A2 provisionals with two different composite resin substrates (A2 and A3) and statistical analysis.

The Δ*E_ab_* values tend to rise with increasing material thickness. This trend is consistent across both types of materials (Telio CAD and Vita CAD-Temp) and both composite resin substrates (A2 and A3). For both A2 and A3 substrates, Telio CAD shows higher Δ*E_ab_* values compared to Vita CAD-Temp at all thicknesses. Telio CAD has a greater color difference when paired with the composite resin substrates than Vita CAD-Temp.

**Figure 2:**
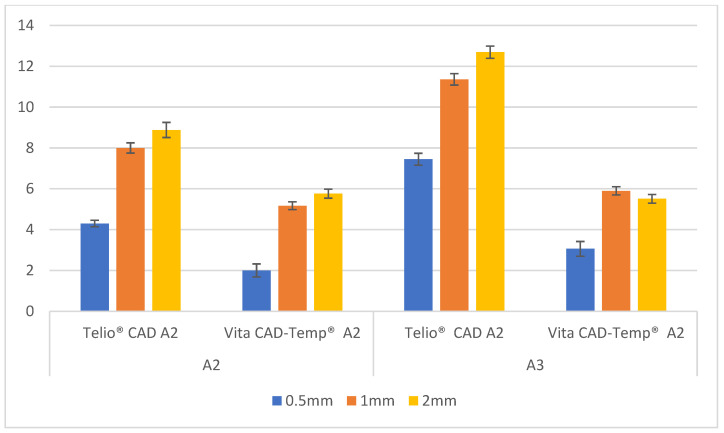
Δ*E_ab_* mean values ± standard deviation (SD) of paired sample groups.

Similar to Table 2, Figure 2 easily demonstrates the Δ*E_ab_* values generally increase as the thickness of the material increases. Telio CAD has a greater color difference when paired with the composite resin substrates compared to Vita CAD-Temp, as it shows higher Δ*E_ab_* values compared to Vita CAD-Temp in all thicknesses. The darker A3 substrate results in greater color change when covered by the provisional material compared to the lighter A2 substrate, as Δ*E_ab_* values are higher for the A3 substrate across all thicknesses and materials.

Levene’s test was used to assess the homogeneity of variances. The test results indicated that variances were equal across the groups, validating the use of ANOVA for the data analysis. ANOVA results showed significant main effects for all three independent variables, indicating that each factor independently affects the Δ*E_ab_* values. Significant interaction effects between the variables were also observed. The impact of one independent variable on Δ*E_ab_* depends on the levels of the other variables. For instance, the difference in Δ*E_ab_* values between Telio CAD and Vita CAD-Temp varied depending on the substrate color and material thickness.

Post-hoc comparisons were conducted using the Bonferroni method to explore these interactions further. It revealed that increasing thickness significantly increased Δ*E_ab_* values for both materials, with Telio CAD showing greater changes than Vita CAD-Temp at each thickness level. Darker substrates (A3) consistently resulted in higher Δ*E_ab_* values compared to lighter substrates (A2) across all material types and thicknesses. This finding underscores the significant impact of substrate color on the final appearance of the restoration. All statistical tests yielded *p*-values less than 0.05. This statistical significance confirms that the differences in color change (Δ*E_ab_*) between Telio CAD and Vita CAD-Temp are not due to random variation but are indeed significant and clinically relevant.

**Figure 3:**
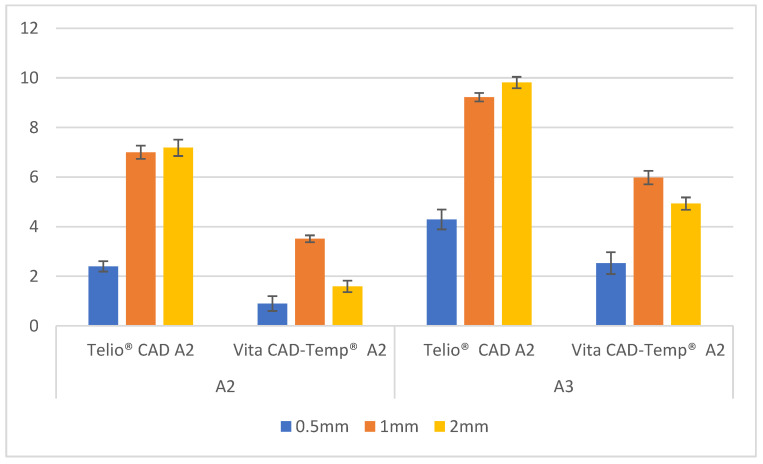
ΔL* mean values ± standard deviation (SD) of paired sample groups.

The variation in lightness (ΔL*) for Telio CAD and Vita CAD-Temp materials was analyzed across different thicknesses (0.5 mm, 1 mm, and 2 mm) and for the two substrates, A2 and A3. These results are presented in Figure 3.

For the A2 substrate, the Telio CAD material exhibited a significant increase in the variation of lightness with increasing thickness. The ΔL* values began at 2.40 ± 0.21 for the 0.5 mm thickness, rising to 7.00 ± 0.27 at 1 mm and reaching 7.18 ± 0.33 at 2 mm. This indicates that the material becomes considerably less light as the thickness increases, especially at greater thicknesses. In contrast, Vita CAD-Temp showed a less pronounced variation in lightness with increasing thickness. The ΔL* values started at 0.90 ± 0.30 for 0.5 mm, increased to 3.51 ± 0.14 at 1 mm, and then decreased to 1.59 ± 0.23 at 2 mm, suggesting a variation in lightness that is not as consistent as observed in Telio CAD.

For the darker A3 substrate, the Telio CAD also demonstrated a significant variation in lightness with increasing thickness. The ΔL* values started at 4.29 ± 0.40 for 0.5 mm, rising to 9.22 ± 0.17 at 1 mm and reaching 9.81 ± 0.23 at 2 mm, showing that the material becomes significantly less light with increasing thickness. On the other hand, Vita CAD-Temp showed a similar pattern to the A2 substrate, with ΔL* values increasing from 2.53 ± 0.44 at 0.5 mm to 5.98 ± 0.27 at 1 mm and then slightly decreasing to 4.93 ± 0.25 at 2 mm. This indicates that there is also a reduction in lightness with increasing thickness, although the changes are more subtle compared to Telio CAD.

These results indicate that the Telio CAD material, in both substrate colors, tends to exhibit a greater variation in lightness with increasing thickness, particularly on darker substrates, while Vita CAD-Temp shows a more moderate and less consistent variation.

**Figure 4:**
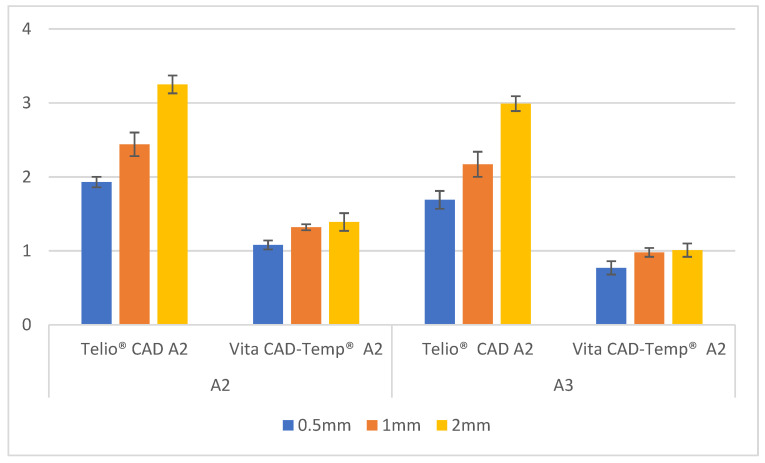
Δa* mean values ± standard deviation (SD) of paired samples groups.

Exploring the variation in the red-green axis (Δa*), detailed in Figure 4, for both materials across different thicknesses (0.5 mm, 1 mm, and 2 mm) and substrates (A2 and A3).

For the A2 substrate, Telio CAD exhibited a clear increase in Δa* values with increasing thickness. The values started at 1.93 ± 0.07 for 0.5 mm thickness and rose to 2.44 ± 0.16 at 1 mm, reaching 3.25 ± 0.12 at 2 mm. This indicates that the material becomes progressively redder as the thickness increases. In contrast, Vita CAD-Temp showed a smaller and more gradual increase in Δa* values with increasing thickness. The values increased from 1.08 ± 0.06 at 0.5 mm to 1.32 ± 0.04 at 1 mm and then slightly to 1.39 ± 0.12 at 2 mm, indicating a much less pronounced shift towards red.

When analyzing the A3 substrate, the Telio CAD material continued to show an increase in Δa* values with increasing thickness, though the values were slightly lower than those observed with the A2 substrate. The Δa* values began at 1.69 ± 0.12 for 0.5 mm, increased to 2.17 ± 0.17 at 1 mm, and reached 2.99 ± 0.10 at 2 mm. This indicates a consistent trend of increased redness with greater thickness, albeit at a slightly lower magnitude than in the A2 substrate. The Vita CAD-Temp material, on the other hand, displayed even smaller changes in Δa* with increasing thickness, with values ranging from 0.77 ± 0.09 at 0.5 mm to 0.98 ± 0.06 at 1 mm and 1.01 ± 0.09 at 2 mm, indicating a minimal shift towards red across all thicknesses.

These results suggest that Telio CAD exhibits a more substantial increase in redness (Δa*) with increasing thickness compared to Vita CAD-Temp, particularly in the A2 substrate. The darker A3 substrate results in a slightly lower, but still noticeable, increase in redness for Telio CAD, while Vita CAD-Temp maintains a more stable red-green balance across varying thicknesses.

**Figure 5:**
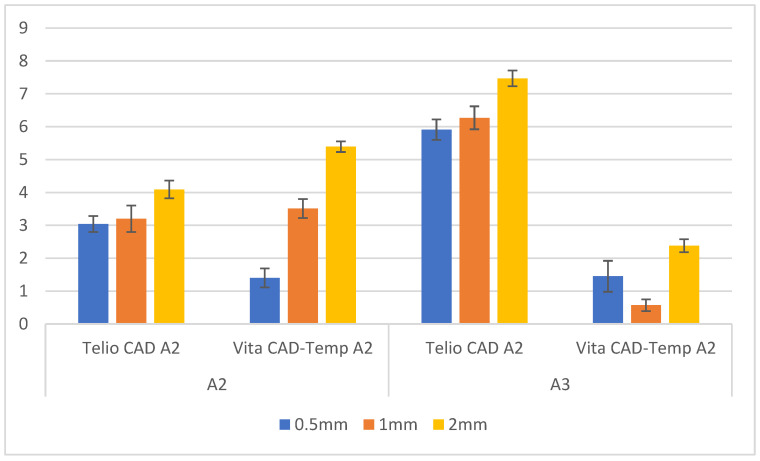
Δb* mean values ± standard deviation (SD) of paired sample groups.

The variation in the yellow-blue axis (Δb*) for both materials and thicknesses and substrates was also scrutinized, and the results are presented in Figure 5.

For the A2 substrate, Telio CAD demonstrated a moderate increase in Δb* values with increasing thickness. The values started at 3.04 ± 0.24 for 0.5 mm thickness, slightly increased to 3.20 ± 0.40 at 1 mm, and then rose more significantly to 4.09 ± 0.27 at 2 mm. This indicates that the material shifts towards the yellow spectrum as the thickness increases. Conversely, Vita CAD-Temp showed a more pronounced increase in Δb* values with thickness, starting at 1.40 ± 0.29 for 0.5 mm, rising to 3.51 ± 0.29 at 1 mm, and reaching 5.39 ± 0.16 at 2 mm. This suggests that Vita CAD-Temp undergoes a more significant yellow shift with increasing thickness compared to Telio CAD on the A2 substrate.

For the A3 substrate, the Telio CAD material exhibited a stronger increase in Δb* values as the thickness increased. The values started at 5.91 ± 0.31 for 0.5 mm, increased to 6.27 ± 0.35 at 1 mm, and reached 7.47 ± 0.24 at 2 mm, indicating a substantial shift towards the yellow spectrum with increased thickness. In contrast, Vita CAD-Temp presented a less consistent trend, with Δb* values starting at 1.45 ± 0.47 for 0.5 mm, decreasing to 0.57 ± 0.18 at 1 mm, and then increasing again to 2.38 ± 0.20 at 2 mm. This variation suggests that Vita CAD-Temp behaviour in terms of yellow-blue shift is less predictable on the darker A3 substrate.

Overall, these results indicate that Telio CAD tends to show a consistent increase in yellowness (Δb*) with increasing thickness, particularly on the A3 substrate, while Vita CAD-Temp exhibits a more variable pattern. The A3 substrate enhances the yellow shift in Telio CAD more noticeably compared to the A2 substrate, whereas Vita CAD-Temp shows a stronger yellow shift on the A2 substrate.

## 4. Discussion

The aesthetic concerns of patients seeking dental treatments have grown significantly in recent times, with the appearance and color of teeth being of utmost importance. Dentistry faces a major hurdle in trying to replicate the visual characteristics of natural teeth using artificial materials. To achieve optimal and durable aesthetic outcomes, it is vital to have a comprehensive understanding of the optical properties of dental materials used in various types of aesthetic restorations [25,26,27]. However, achieving the desired aesthetic outcome with ceramic restorations depends not only on the material selection but also on proper tooth preparation, choice of resin cement, and surface treatments [28,29]. The color of the underlying tooth or the base where the ceramic restoration will be placed, as well as the cement used, can also impact the overall result [30,31,32,33].

The findings of this study are highly significant for clinicians as they directly impact the aesthetic outcomes of dental restorations, which are critical for patient satisfaction. Color mismatches, even subtle ones, can lead to noticeable discrepancies between the restoration and natural teeth, potentially resulting in patient dissatisfaction and a lack of confidence in the treatment. These mismatches can also necessitate additional corrective procedures, increasing treatment time and costs. By understanding how different provisional materials and thicknesses influence color stability, clinicians can make more informed decisions, ensuring better aesthetic integration and enhancing overall clinical outcomes.

The rationale behind choosing the thicknesses of 0.5 mm, 1.0 mm, and 2.0 mm in this study is grounded in their relevance to clinical practice, particularly in the fabrication of dental restorations. These thicknesses represent the range typically encountered in provisional restorations, where material thickness significantly influences both the aesthetic and functional outcomes. A 0.5 mm thickness is commonly used in areas where minimal material is required to maintain tooth structure, such as in thin anterior veneers or when preserving natural teeth is critical. It tests the material’s ability to mask the underlying tooth color while remaining thin. An intermediate thickness, 1.0 mm, is representative of many standard provisional restorations. It balances strength and aesthetics, making it a common choice in clinical settings. It provides insight into how the material performs under typical conditions where moderate masking ability is needed. A much thicker option is often used in cases requiring significant masking of underlying tooth discoloration or in situations where additional material thickness is necessary for structural integrity or when replacing old metal ceramic crowns. It tests the material’s ability to maintain color stability and aesthetic appearance when more substantial coverage is required.

By evaluating these specific thicknesses, the study aims to provide practical insights into how different provisional materials perform across a range of clinically relevant scenarios, helping clinicians choose the most appropriate thickness for achieving optimal aesthetic and functional outcomes.

According to the results obtained in this study, the color difference (Δ*E_ab_*) between the initial composite resin sample and the sample after the addition of a provisional material is consistently perceptible (>1.2) and falls within the “unacceptable” range (>2.7). This finding holds true regardless of the color of the base, the type of material, or the thickness used. This indicates that the addition of any provisional material leads to a noticeable and clinically significant change in color, which, depending on the clinical situation, does or does not meet the accepted aesthetic standards.

In the context of dental restorations, perceptibility and acceptability thresholds are critical metrics used to evaluate color differences. A Δ*E_ab_* value greater than 1.2 indicates that the color difference is perceptible to the human eye under normal viewing conditions. When this difference exceeds 2.7, it is deemed “unacceptable”, meaning it is large enough to be noticeable and potentially problematic for the patient’s satisfaction with the aesthetic outcome.

The findings from this study are particularly relevant when considering the aesthetic demands of dental patients. In modern dentistry, especially in visible areas of the mouth, achieving a color match that is indistinguishable from the surrounding natural teeth is paramount. The fact that all tested combinations in this study resulted in Δ*E_ab_* values exceeding the perceptibility and acceptability thresholds highlights a significant challenge in the use of provisional materials.

The study further reveals that the base color significantly influences the final color of the composite resin and provisional material combination. Specifically, when using an A3 base, the observed Δ*E* value is consistently higher compared to an A2 base. This suggests that darker base colors, such as A3, exacerbate the color difference when a provisional material is added, leading to a more pronounced and often unacceptable change in appearance. Darker bases/teeth tend to absorb more light and alter the way light interacts with the provisional material, thus enhancing the color shift.

Previous studies have extensively investigated the impact of substrate color, material type, and thickness on the final color of dental restorations, particularly through the use of the CIELAB color system [21,22,34,35]. Research in this area has demonstrated that the substrate color, which refers to the underlying tooth or restorative material, plays a pivotal role in determining the overall appearance of the final restoration. This is particularly important in aesthetic zones where even slight color discrepancies can be easily detected by patients [21,22,34,35]. We believe that ours is the first to address this with long-term provisionals.

Previous research has primarily focused on permanent restorative materials; this study provides new insights into the performance of CAD/CAM provisional materials [19,20,21,22]. The finding that Telio CAD exhibits greater color changes than Vita CAD-Temp across all thicknesses is a novel contribution, offering clinicians specific guidance on material selection based on aesthetic needs. This is particularly relevant for temporary restorations, where the balance between aesthetic appearance and practical functionality is critical.

Darker substrates (A3) result in higher Δ*E_ab_* values across both materials and all thicknesses. This underscores the significant impact that substrate color has on the final appearance of the restoration. In clinical practice, this means that the color of the underlying tooth or composite base must be carefully considered, as it can markedly affect the visual outcome.

For aesthetic applications and when biomimetic principles dictate the preservation of tooth structure, a provisional material that can achieve significant color change with minimal thickness should be considered. Telio CAD is effective in these scenarios due to its higher Δ*E_ab_* values, which enable it to mask the underlying substrate color more effectively, even at reduced thicknesses. Conversely, Vita CAD-Temp might be preferred when less color change is needed and a more subtle effect is desired, as it exhibits lower Δ*E_ab_* values, indicating less color change.

While Telio CAD demonstrates excellent color masking capabilities with higher Δ*E_ab_* values, clinicians must also consider its mechanical properties. The inferior fracture load and surface wear resistance of materials such as Vita CAD-Temp, as evidenced by Hensel et al. [36], suggest that a balanced approach is necessary. Thus, an integrated assessment of both aesthetic and mechanical properties is crucial for making informed clinical decisions.

The analysis of the L* (lightness), a* (green-red axis), and b* (blue-yellow axis) components provides valuable insights into how provisional materials interact with underlying substrates. Telio CAD demonstrates a notable increase in lightness (L*) with increasing thickness, especially on darker substrates, suggesting that thicker Telio CAD layers may effectively mask darker underlying substrates. This effect is less pronounced in the Vita CAD-Temp material, suggesting that its composition, including possible differences in filler content or pigment types, interacts differently with the substrate’s color [37,38].

The increase in lightness observed with greater material thicknesses resulted in perceptible color differences in many cases. Specifically, the Telio CAD A2 material exceeded the acceptability threshold (ΔL* > 2.7) at both 1 mm and 2 mm thicknesses on both the A2 and A3 substrates, indicating that the brightness change was not only noticeable but also potentially unacceptable from an aesthetic standpoint. In contrast, the Vita CAD-Temp A2 material generally remained within acceptable limits, except when applied over the A3 substrate at 1 mm and 2 mm thicknesses, where the ΔEab values also surpassed the 2.7 threshold, suggesting a similar concern for aesthetic integration.

Additionally, Telio CAD exhibits a consistent shift towards green (a*) as thickness increases, contributing to overall color differences. Pecho et al. [39] similarly observed that the a* values for Filtek™ Supreme A2 composite resin, measured at a 1 mm thickness, were lower (indicating a more greenish hue) when compared to the Filtek™ Supreme A3 base. The discrepancies between our findings and those of Pecho et al. [39] could be attributed to variations in the measurement conditions, such as the background color used during measurements (black versus white) and the type of instrument employed (spectroradiometer). This highlights the importance of substrate color and the specific measurement setup in determining the red-green color shift.

The decrease in a* values, indicating a shift towards a greener hue, was also found to be significant in certain scenarios. The Telio CAD material once again showed a trend where the Δa* exceeded the acceptability threshold at 1 mm and 2 mm thicknesses on both substrates, making these shifts in the red-green axis potentially problematic for color matching in clinical practice. However, for the Vita CAD-Temp material, the shifts in a* were generally perceptible (Δa* > 1.2) but stayed within the acceptable range (Δa* < 2.7), particularly when applied over the A2 substrate. This indicates that while there was a noticeable color change, it was less likely to be deemed unacceptable by patients.

The decrease in yellowness (b*) with thickness for both Telio CAD and Vita CAD-Temp indicates potential color adjustments, with Telio CAD showing a more substantial blue shift, which is beneficial for colder tones. Variations in the b* coordinate between different composite resin shades are influenced by differences in the materials’ composition and the pigments used in their formulation. Composite resins typically contain a blend of organic resins, inorganic fillers, and specific pigments to achieve the desired color and translucency for dental restorations. Inorganic oxides, such as ferrous oxide for red tones and ferric hydroxide for yellow, are often added in small amounts to help create shades that closely resemble natural teeth [37,39]. Consistent with our findings, previous research, including a study by Pecho et al. [39], has shown that the b* values tend to be higher for A3 composite resin bases compared to A2. This pattern was observed with the same brand and shades of composite resin as those used in our study, suggesting that the composition and pigmentation are key factors driving these differences in the yellow-blue axis. Such findings highlight the importance of carefully selecting materials based on their color properties to achieve the desired aesthetic outcomes in dental restorations.

The decrease observed in b* values, which corresponds to a shift towards a more bluish tone, also has significant implications for color perception. The Telio CAD material demonstrated Δb* values that exceeded the acceptability threshold at 1 mm and 2 mm thicknesses on both the A2 and A3 substrates. This suggests that the yellow-blue shift could be highly noticeable and potentially unsatisfactory in these cases. The Vita CAD-Temp material, on the other hand, remained mostly within the acceptable range on the A2 substrate but showed unacceptable color differences (Δb* > 2.7) at 2 mm thickness when applied over the A3 substrate.

Significant shifts in the L*, a*, and b* values of Telio CAD suggest its responsiveness to thickness and substrate color variations, offering versatility but also posing challenges in clinical contexts. In contrast, Vita CAD-Temp demonstrates more stable color behavior, suitable for cases requiring minimal color change [40]. These findings underscore the importance of considering the individual contributions of L*, a*, and b* to overall color differences, which vary based on material and substrate combinations. The pronounced shifts in the L*, a*, and b* values of Telio CAD suggest its sensitivity to changes, while Vita CAD-Temp’s consistent color balance offers advantages in scenarios of subtle adjustments.

Collectively, our results indicate that the variations in Δa* and Δb* Vita CAD-Temp make Vita CAD Temp less predictable than when using Telio CAD. However, the thickness is the most relevant factor for Vita CAD-Temp. Understanding how these provisional materials interact with substrates and how their color properties evolve with thickness is crucial for achieving desired aesthetic outcomes in clinical practice.

Given that all *p*-values are less than 0.05, we reject the null hypothesis. This statistical significance indicates that there are indeed significant differences in the color change (Δ*E_ab_*) between Telio CAD and Vita CAD-Temp across different material thicknesses and substrate colors.

The study sheds light on the unique color behavior of different provisional materials, particularly in how Telio CAD shows more substantial shifts in lightness (L*), redness (a*), and yellowness (b*) compared to Vita CAD-Temp. This differentiation between materials adds a new dimension to the understanding of how provisional materials interact with substrate colors and thicknesses, which has not been extensively explored in the literature.

## 5. Conclusions

Clinicians should consider the specific requirements of the restoration and the underlying substrate color when selecting the material and thickness. Thinner layers of Telio CAD will produce more noticeable color changes, which could be advantageous or detrimental depending on the clinical scenario.

The primary objective of our research is to improve clinical practices. We conclude that when the substrate color is darker, using a thicker provisional layer is advisable. This recommendation aims to enhance the brightness and aesthetic appeal of the final restoration, ultimately improving the overall outcome of the procedure. By considering this factor, clinicians can achieve more visually pleasing results in their dental work.

Nonetheless, when using Telio CAD, less tooth preparation is required to achieve the same level of color change compared to Vita CAD-Temp. This is crucial for maintaining tooth integrity and following biomimetic principles. For achieving noticeable color changes and optimal aesthetic results with minimal material thickness, Telio CAD is preferable. However, for more conservative color adjustments, Vita CAD-Temp may be the better choice.

However, it is important to note that further research and clinical studies are necessary to validate and expand upon these findings. Additionally, other factors such as patient preferences, oral conditions, and individual case considerations should also be taken into account when determining the optimal thickness of the restorative materials.

## Figures and Tables

**Table 1:** Materials used in the study.

Material and Manufacturer	Composition	Batch Number
Telio^®^ CAD (Ivoclar Vivadent. Schaan, Liechtenstein) LT BL3 A2	>98% ester-based cross-linked polymethyl methacrylate, <0.1% pigments	Z04CK9
VITA CAD-Temp^®^ (VITA Zahnfabrik, Bad Säckingen, Germany) 2 M2T A2	83–86% poly-methyl methacrylate (PMMA); 14% SiO_2_; <0.1% pigments	99510
Filtek^TM^ Supreme XTE Universal Restorative Body Shade A2 (3M ESPE, St. Paul, MN, USA)	UDMA, TEGMA, Bis-GMA, Bis-EMA, silica (20 nm), Zirconia, pigments. (4–11 nm). Average particle size 0.6 to 10 μm. Inorganic particles represent 78.5% of the total charge.	NF22572
Filtek^TM^ Supreme XTE Universal Restaurative Body Shade A3 (3M ESPE, St. Paul, MN, USA)	UDMA, TEGMA, Bis-GMA, Bis-EMA, silica (20 nm), Zirconia, pigments. (4–11 nm). Average particle size 0.6 to 10 μm. Inorganic particles represent 78.5% of the total charge.	N927242

**Table 2:** Δ*E_ab_* mean values ± standard deviation (SD) of paired sample groups. The same letters represent a direct comparison between the groups.

		Thicknesses			
Bases	Material	0.5 mm	1 mm	2 mm	
A2	Telio CAD A2	4.30 ± 0.16	8.00 ± 0.25	8.88 ± 0.37	*p* < 0.001 ^(a)(^*^)^
	Vita CAD-Temp A2	2.00 ± 0.32	5.17 ± 0.19	5.76 ± 0.22	*p* < 0.001 ^(a)(^*^)^
A3	Telio CAD A2	7.45 ± 0.29	11.36 ± 0.28	12.69 ± 0.30	*p* < 0.001 ^(a)(^*^)^
	Vita CAD-Temp A2	3.06 ± 0.36	5.90 ± 0.20	5.51 ± 0.21	*p* < 0.001 ^(a)(^*^)^
		*p* < 0.001 ^(a)(^*^)^	*p* < 0.001 ^(a)(^*^)^	*p* < 0.001 ^(a)(^*^)^	

(a) Three-way mixed ANOVA; (*) Identifies a statistically significant difference for a 95% confidence interval.

## Data Availability

The data presented in this study are available on request from the corresponding author.

## References

[1] Joiner A. (2004). Tooth Colour: A Review of the Literature. J. Dent..

[2] Jahangiri L., Reinhardt S.B., Mehra R.V., Matheson P.B. (2002). Relationship between Tooth Shade Value and Skin Color: An Observational Study. J. Prosthet. Dent..

[3] Sikri V.K. (2010). Color: Implications in dentistry. J. Conserv. Dent..

[4] Kim H.-K. (2018). Evaluation of the Repeatability and Matching Accuracy between Two Identical Intraoral Spectrophotometers: An in Vivo and in Vitro Study. J. Adv. Prosthodont..

[5] El Mourad A.M., Al Shamrani A., Al Mohaimeed M., Al Sougi S., Al Ghanem S., Al Manie W. (2021). Self-Perception of Dental Esthetics among Dental Students at King Saud University and Their Desired Treatment. Int. J. Dent..

[6] Ten Bosch J.J., Coops J.C. (1995). Tooth Color and Reflectance as Related to Light Scattering and Enamel Hardness. J. Dent. Res..

[7] de Menezes R.P., Silva P.D., Leal P.C., Faria-e-Silva A.L. (2018). Impact of 35% Hydrogen Peroxide on Color and Translucency Changes in Enamel and Dentin. Braz. Dent. J..

[8] He W., Park C.J., Byun S., Tan D., Lin C.Y., Chee W. (2020). Evaluating the Relationship between Tooth Color and Enamel Thickness, Using Twin Flash Photography, Cross-polarization Photography, and Spectrophotometer. J. Esthet. Restor. Dent..

[9] Spitznagel F.A., Boldt J., Gierthmuehlen P.C. (2018). CAD/CAM Ceramic Restorative Materials for Natural Teeth. J. Dent. Res..

[10] Chaiyabutr Y., Kois J.C., Lebeau D., Nunokawa G. (2011). Effect of Abutment Tooth Color, Cement Color, and Ceramic Thickness on the Resulting Optical Color of a CAD/CAM Glass-Ceramic Lithium Disilicate-Reinforced Crown. J. Prosthet. Dent..

[11] Wingo K. (2018). A Review of Dental Cements. J. Vet. Dent..

[12] Archegas L.R.P., Freire A., Vieira S., de Menezes Caldas D.B., Souza E.M. (2011). Colour Stability and Opacity of Resin Cements and Flowable Composites for Ceramic Veneer Luting after Accelerated Ageing. J. Dent..

[13] Chang J., Da Silva J.D., Sakai M., Kristiansen J., Ishikawa-Nagai S. (2009). The Optical Effect of Composite Luting Cement on All Ceramic Crowns. J. Dent..

[14] Liberato W.F., Barreto I.C., Costa P.P., de Almeida C.C., Pimentel W., Tiossi R. (2019). A Comparison between Visual, Intraoral Scanner, and Spectrophotometer Shade Matching: A Clinical Study. J. Prosthet. Dent..

[15] Edelhoff D., Beuer F., Schweiger J., Brix O., Stimmelmayr M., Guth J.-F. (2012). CAD/CAM-Generated High-Density Polymer Restorations for the Pretreatment of Complex Cases: A Case Report. Quintessence Int..

[16] Pietrobon N., Lehner C.R., Schärer P. (1996). Long-Term Temporary Dentures in Crown and Bridge Prosthesis. The Design Principles, Choice of Material and Practical Procedure. Schweiz. Monatsschr. Zahnmed..

[17] LeSage B.P. (2020). CAD/CAM: Applications for Transitional Bonding to Restore Occlusal Vertical Dimension. J. Esthet. Restor. Dent..

[18] Burian G., Erdelt K., Schweiger J., Keul C., Edelhoff D., Güth J.-F. (2021). In-Vivo-Wear in Composite and Ceramic Full Mouth Rehabilitations over 3 Years. Sci. Rep..

[19] Paravina R.D., Ghinea R., Herrera L.J., Bona A.D., Igiel C., Linninger M., Sakai M., Takahashi H., Tashkandi E., Mar Perez M. (2015). del Color Difference Thresholds in Dentistry. J. Esthet. Restor. Dent..

[20] Sampaio C.S., Belfus J., Avila A., Cordero C., Freitte M., Ferrari V., Atria P.J., Jorquera G. (2021). Effect of Different Fabrication Steps on Color and Translucency of a CAD-CAM Feldspathic Ceramic. J. Esthet. Restor. Dent..

[21] Gomes C., Martins F., Reis J.A., Maurício P.D., Ramírez-Fernández M.P. (2023). Color Assessment of Feldspathic Ceramic with Two Different Thicknesses, Using Multiple Polymeric Cements. Polymers.

[22] Pereira J.S.D.C., Reis J.A., Martins F., Maurício P., Fuentes M.V. (2022). The Effect of Feldspathic Thickness on Fluorescence of a Variety of Resin Cements and Flowable Composites. Appl. Sci..

[23] Carrabba M., Vichi A., Tozzi G., Louca C., Ferrari M. (2020). Cement Opacity and Color as Influencing Factors on the Final Shade of Metal-free Ceramic Restorations. J. Esthet. Restor. Dent..

[24] Bayindir F., Koseoglu M. (2020). The Effect of Restoration Thickness and Resin Cement Shade on the Color and Translucency of a High-Translucency Monolithic Zirconia. J. Prosthet. Dent..

[25] Alayad A.S., Alqhatani A., Alkatheeri M.S., Alshehri M., AlQahtani M.A., Osseil A.E.B., Almusallam R.A. (2021). Effects of CAD/CAM Ceramics and Thicknesses on Translucency and Color Masking of Substrates. Saudi Dent. J..

[26] Araujo E., Perdigão J. (2021). Anterior Veneer Restorations—An Evidence-Based Minimal-Intervention Perspective. J. Adhes. Dent..

[27] Joiner A., Luo W. (2017). Tooth Colour and Whiteness: A Review. J. Dent..

[28] Günal-Abduljalil B., Ulusoy M.M. (2022). The Effect of Resin Cement Shade and Restorative Material Type and Thickness on the Final Color of Resin-Matrix Ceramics. J. Prosthodont. Res..

[29] de Matos J.D.M., Nakano L.J.N., Bottino M.A., de Jesus R.H., Maciel L.C. (2020). Current Considerations for Dental Ceramics and Their Respective Union Systems. Rev. Bras. Odontol..

[30] Comba A., Paolone G., Baldi A., Vichi A., Goracci C., Bertozzi G., Scotti N. (2022). Effects of Substrate and Cement Shade on the Translucency and Color of CAD/CAM Lithium-Disilicate and Zirconia Ceramic Materials. Polymers.

[31] Dede D.Ö., Ceylan G., Yilmaz B. (2017). Effect of Brand and Shade of Resin Cements on the Final Color of Lithium Disilicate Ceramic. J. Prosthet. Dent..

[32] Șoim A., Strîmbu M., Burde A.V., Culic B., Dudea D., Gasparik C. (2018). Translucency and Masking Properties of Two Ceramic Materials for Heat-Press Technology. J. Esthet. Restor. Dent..

[33] Sonza Q.N., Della Bona A., Pecho O.E., Borba M. (2021). Effect of Substrate and Cement on the Final Color of Zirconia-Based All-Ceramic Crowns. J. Esthet. Restor. Dent..

[34] Pires L.A., Novais P.M.R., Araújo V.D., Pegoraro L.F. (2017). Effects of the Type and Thickness of Ceramic, Substrate, and Cement on the Optical Color of a Lithium Disilicate Ceramic. J. Prosthet. Dent..

[35] Niu E., Agustin M., Douglas R.D. (2014). Color Match of Machinable Lithium Disilicate Ceramics: Effects of Cement Color and Thickness. J. Prosthet. Dent..

[36] Hensel F., Koenig A., Doerfler H.-M., Fuchs F., Rosentritt M., Hahnel S. (2021). CAD/CAM Resin-Based Composites for Use in Long-Term Temporary Fixed Dental Prostheses. Polymers.

[37] Klapdohr S., Moszner N. (2005). New Inorganic Components for Dental Filling Composites. Monatshefte Für Chem. Chem. Mon..

[38] Lim Y.-K., Lee Y.-K., Lim B.-S., Rhee S.-H., Yang H.-C. (2008). Influence of Filler Distribution on the Color Parameters of Experimental Resin Composites. Dent. Mater..

[39] Pecho O.E., Pérez M.M., Ghinea R., Della Bona A. (2016). Lightness, Chroma and Hue Differences on Visual Shade Matching. Dent. Mater..

[40] Pop-Ciutrila I.-S., Ghinea R., Colosi H.A., Ruiz-López J., Perez M.M., Paravina R.D., Dudea D. (2021). Color Compatibility between Dental Structures and Three Different Types of Ceramic Systems. BMC Oral Health.

